# Intracystic Papillary Carcinoma: A Case Report

**DOI:** 10.7759/cureus.28504

**Published:** 2022-08-28

**Authors:** Brittany L Miles, Jing He, Quan D Nguyen

**Affiliations:** 1 Medical Education, University of Texas Medical Branch, Galveston, USA; 2 Pathology, University of Texas Medical Branch, Galveston, USA; 3 Radiology, Baylor College of Medicine, Houston, USA

**Keywords:** diagnosis & prognosis, papillary dcis, papillary breast lesions, in-situ carcinoma, intracystic papillary carcinoma

## Abstract

Intracystic papillary carcinoma (IPC) of the breast is a rare form of in-situ carcinoma, which is contained within a dilated duct. Mammography and ultrasound may provide clues to its presence, but formal diagnosis always requires histologic evidence. Although IPC is associated with an excellent prognosis, surgical resection is important in order to rule out the possibility of any invasive component, which would result in the need for more aggressive treatment. In this paper, we review the radiographic and histologic features of this interesting diagnosis, present a patient case, and explore the possible reason why IPC does not require the same treatment modalities as the more common ductal carcinoma in situ (DCIS).

## Introduction

Intracystic papillary carcinoma (IPC) is a rare malignancy, occurring in approximately 0.5% of breast carcinoma cases [1]. It is defined as a solitary tumor with a histologic pattern consistent with in-situ carcinoma but confined within a dilated duct [2]. Intracystic papillary carcinomas usually arise from the wall of macrocysts of the breast and pathologically demonstrate cellular fronds supported by a fibrovascular stalk [3,4]. Preoperative evaluation is performed by mammography, ultrasonography, pneumocystography, and aspiration cytology [5,6].

Mammography will typically show a sharply circumscribed cystic mass, sometimes with an irregular or nodular contour. If the border appears shaggy, it may indicate invasion through the cyst wall into the surrounding parenchyma [7]. Color Doppler may be capable of demonstrating intramural blood flow in the mass portion of the lesion and provide an early indication of the diagnosis [4].

## Case presentation

**Figure 1:**
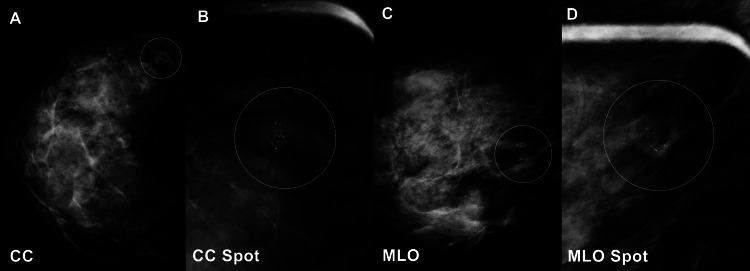
The right breast contains a 17 mm oval mass with circumscribed margins and punctate and indistinct calcifications (A) The craniocaudal (CC), (B) CC spot compression, (C) mediolateral oblique (MLO), and (D) MLO spot compression views are presented.

A 51-year-old woman was found to have an abnormality on her initial screening mammogram. She denied any symptoms attributable to the lesion. The physical exam did not reveal any mass, skin dimpling, nipple retraction, or nipple discharge. Her mammogram of the right breast revealed a 17 mm oval mass in the lower outer quadrant that was associated with punctate and indistinct calcifications (Figure 1).

**Figure 2:**
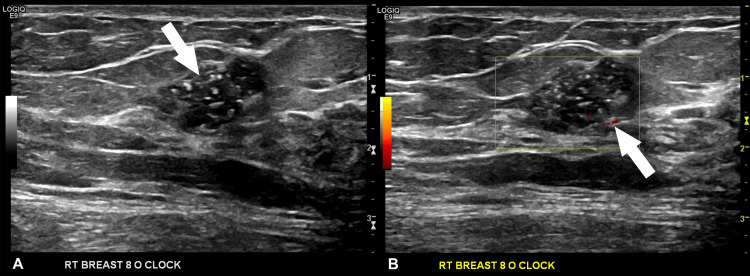
(A) Ultrasound shows an 18 x 10 mm solid mass at the 8 o’clock position of the right breast; (B) Internal calcifications are seen, and Doppler analysis shows vascularity with a small amount of blood flow in the periphery

Ultrasound of the area showed a microlobulated solid lesion associated with microcalcifications, with an orientation parallel to the skin. Color Doppler revealed the presence of vascularity within the wall of the lesion and a core biopsy was recommended (Figure 2).

**Figure 3:**
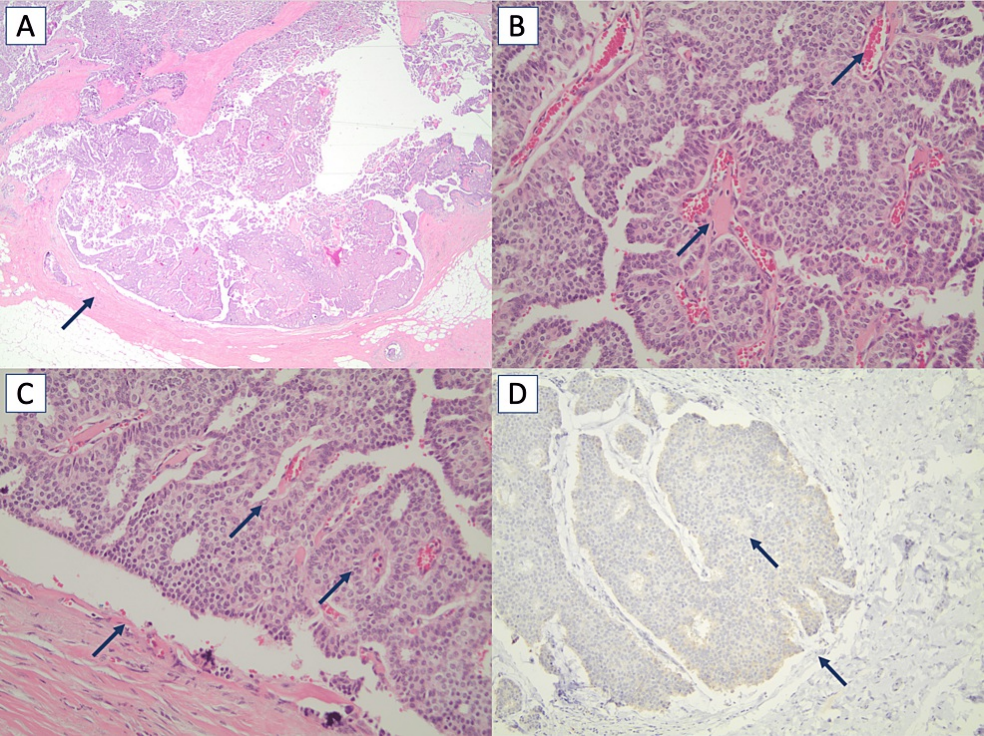
Histological findings of lumpectomy of the breast mass (A) Hematoxylin and eosin (H&E) staining in low magnification reveals a thick fibrous capsule surrounding the nodular lesion with papillary fronds (magnification: 20x). (B) The nodule is composed of fibrovascular cores covered by monomorphic neoplastic cells arranged in solid or cribriform patterns (magnification: 200x). (C) Myoepithelial cells are absent both within the fibrovascular cores and the peripheral of the lesion (magnification: 200x). (D) p63 immunostaining demonstrates the absence of myoepithelial cells both within and at the periphery of the lesion (magnification: 100x).

Intracystic papillary carcinoma was confirmed on the initial core biopsy. Because the breast lesion was not detectable on physical exam, preoperative needle localization was performed under imaging guidance with a J-shaped wire, and a postoperative x-ray of the lesion provided radiographic confirmation of resection. Pathologic analysis was then performed to ensure that all margins were free of involvement. Analysis of the resected mass revealed a 12 mm intracystic papillary carcinoma with central necrosis (Figure 3).

No lymphovascular or perineural invasion was seen. Estrogen and progesterone receptors were positive in 90% of cells. Following resection, the patient was followed with surveillance for more than five years and did not have any evidence of recurrence.

## Discussion

IPC is a rare and unusual form of carcinoma in situ, which arises from the wall of a macrocyst in the breast. The accumulation of fluid inside the cyst is believed to be the main contributor to the large average size of these lesions at presentation [1]. The fluid obtained by aspiration of IPC is usually hemorrhagic, either because of tumor necrosis or from the rupture of capillaries within the cyst wall [8-10]. One study reported that measurement of the intracystic fluid carcinoembryonic antigen (CEA) level may be useful for diagnosing the presence of malignancy when it is significantly elevated in comparison to the serum level [11]. If fluid analysis and cytology are not helpful in obtaining the diagnosis, the presence of a residual mass after cyst aspiration is an indication of open resection [12].

Nonpalpable breast lesions are preoperatively localized with imaging, which can include mammography, ultrasound, or MRI. Sometimes a radiopaque clip is placed at the time of initial biopsy if malignancy is confirmed and neoadjuvant treatment may make the lesion more difficult to identify in the future. Preoperatively, a J-shaped wire is frequently placed into the breast lesion to make it easier for the surgeon to identify at the time of surgery.

Because it is an in-situ malignancy, it is tempting to compare IPC to the much more common ductal carcinoma in situ (DCIS), which has a different treatment strategy. Studies of IPC indicate that surgical excision is often the only required intervention, with survival rates approaching 100%. This differs significantly from DCIS, for which the addition of radiation and antiestrogen therapies are the standard of care. It would seem logical that the primary reason for this difference in prognosis is that IPC is encapsulated within a dilated tubule, which provides physical containment, and in the absence of any invasive component, none of the carcinoma-in-situ cells are capable of escaping confinement. Invasive carcinoma would need to be present for cells to either escape through the cyst wall or enter the circulation that provides blood supply to the solid papillary carcinoma components within the structure. It is therefore important that an appropriate histological evaluation be performed to ensure that no invasion is present in the specimen.

The prognosis of intrapapillary cystic carcinoma is usually excellent and unrelated to the size of the lesion [7]. When intracystic papillary carcinoma is contained completely within the cyst and there is no associated DCIS or invasion, it can be managed with wide local excision as the only necessary intervention [13]. The presence of any associated DCIS or invasion would necessitate that treatment be directed at the most aggressive histology identified [14]. Low-grade tumors may be treated by local excision, while higher-grade lesions have an increased risk of recurrence or metastasis [15]. There is currently no consensus regarding the management of the axilla in patients with IPC. One study reported a nodal involvement rate of 8%, although the percentage of patients who actually underwent axillary sampling was unknown [16]. For this reason, some authors advocate for sentinel lymph node analysis in this patient population [17-19].

Following surgery, the appropriate schedule for surveillance has not been established although some authors recommend following the same protocol that is advised for DCIS [16]. This would include an interval history and physical exam every 6-12 months for five years and then annually. The initial postoperative mammogram should occur 6-12 months after surgery and then annually [20].

## Conclusions

Intracystic papillary carcinoma of the breast is an interesting and rare version of carcinoma in situ, which is associated with an excellent prognosis. Sometimes, the diagnosis can be suspected based on imaging performed prior to cyst aspiration, but the presence of a bloody aspirate or a residual mass after aspiration should prompt surgical resection regardless of the aspirate cytology. The encapsulation provided by the cyst wall likely provides a physical barrier that allows IPC to be treated differently from DCIS.
